# Synuclein Proteins in Cancer Development and Progression

**DOI:** 10.3390/biom13060980

**Published:** 2023-06-12

**Authors:** Lucía C. Zanotti, Florencia Malizia, Nahuel Cesatti Laluce, Aylén Avila, Macarena Mamberto, Luciano E. Anselmino, Mauricio Menacho-Márquez

**Affiliations:** 1 Instituto de Inmunología Clínica y Experimental de Rosario (IDICER, CONICET-UNR), Facultad de Ciencias Médicas (UNR), Rosario 3100, Argentina; 2 Instituto de Inmunología Clínica y Experimental, CONICET, Rosario 3100, Argentina; 3 Centro de Investigación y Producción de Reactivos Biológicos (CIPReB), Facultad de Ciencias Médicas (UNR), Suipacha 660, Rosario 2000, Argentina; 4 Centro de Investigación del Cáncer de Rosario, Red de Investigación del Cáncer de Rosario (RICaR), 37007 Salamanca, Spain

**Keywords:** synucleins, cancer, aggregation, pathways, biomarkers

## Abstract

Synucleins are a family of small, soluble proteins mainly expressed in neural tissue and in certain tumors. Since their discovery, tens of thousands of scientific reports have been published about this family of proteins as they are associated with severe human diseases. Although the physiological function of these proteins is still elusive, their relationship with neurodegeneration and cancer has been clearly described over the years. In this review, we summarize data connecting synucleins and cancer, going from the structural description of these molecules to their involvement in tumor-related processes, and discuss the putative use of these proteins as cancer molecular biomarkers.

## 1. Introduction

Synucleins are small, highly conserved proteins implicated in neurodegenerative disorders and cancer. This family is composed of three members, alpha, beta, and gamma synuclein (αS, βS, and γS, respectively). Synucleins are commonly described as intrinsically disordered proteins (IDPs), as they lack a fixed or ordered three-dimensional structure, and they contain intrinsically disordered regions that lack secondary structure and global topology [1].

The first member of this family to be discovered was αS, which was isolated from the electric ray Torpedo Californica in 1988 by the use of an antiserum against purified cholinergic synaptic vesicles [2]. The name synuclein was coined because this first study revealed a neuron-specific protein with nuclear and presynaptic terminal localization, which was proposed to be involved in coordinating nuclear and synaptic neuronal events [2]. Discovery of αS was rapidly followed by the identification of two close homologs, βS and γS. βS was first identified in 1990 by Nakajo et al. as a 14 kDa phosphoneuroprotein present in bovine brain [3] and its complete sequence was soon published by the same group [4]. It was in 1994 when αS and βS were purified and sequenced from human brain, and their close homology established the existence of a family of human brain synucleins [5]. The last discovered member of the family was γS, identified as a differentially expressed gene in breast cancer, and it was first named breast cancer-specific gene 1 (BCSG1) because it was abundant in advanced infiltrating breast carcinoma and almost undetectable in normal or benign breast lesions [6]. After cloning from brain genomic and cDNA libraries, the previously identified BCSG1, also called persyn [7,8], was named as SNCG and considered to be the third member of the synuclein family [9].

## 2. Synuclein Structure and Homology

Synucleins are intrinsically disorder or unstructured proteins prone to aggregate, involved in severe human diseases. Around 30% of the eukaryotic proteins contain intrinsically disordered regions lacking secondary structure and global topology, despite representing functional states [10]. This abundancy suggests their importance in key cellular processes such as homeostasis and survival [11]. IDPs are characterized by containing few hydrophobic residues, a high net charge, low sequence complexity, and structure-breaking residues (e.g., proline) that facilitate disorder [12,13].

**Figure 1:**
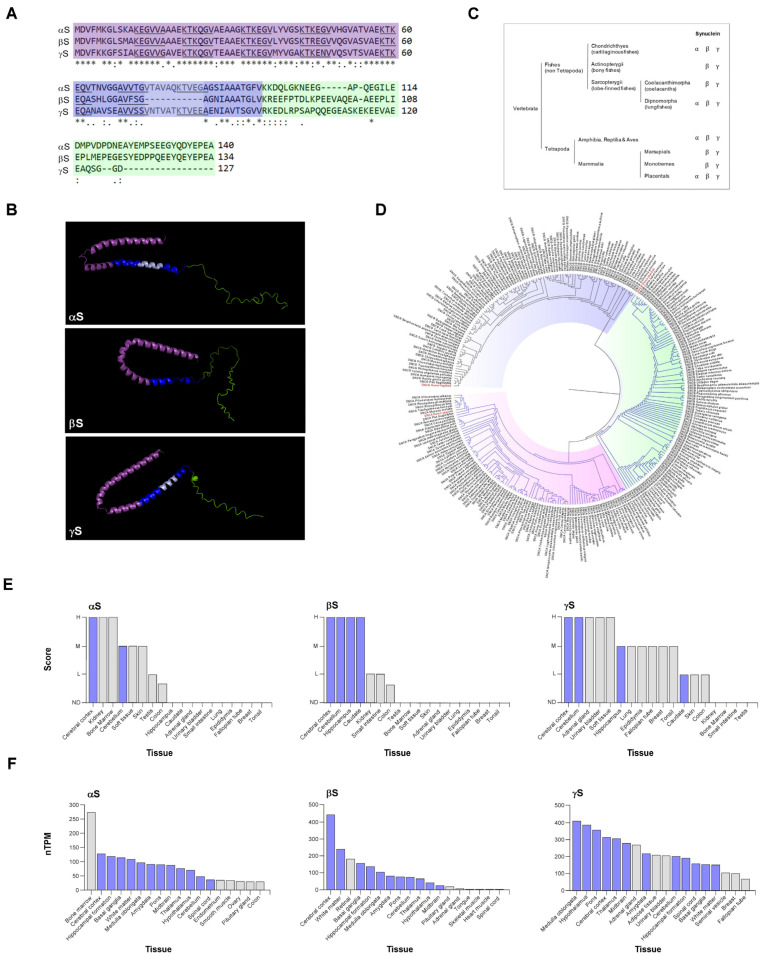
Structure of synucleins, phylogeny, and expression in human tissues. (**A**) Multiple sequence alignment of human synucleins by Clustal W. Sequences were obtained from NCBI (accession numbers CAG33339.1; CAG33308.1 and CAG46587.1). “*” indicates identical amino acids; “:” and “.” indicate conserved and semi-conserved residues, respectively. Each synuclein is organized in a tripartite arrangement, with the N-terminal region (light violet), the central NAC region (dark blue), and the C-terminal region (light green). Amino acids involved in aggregation are marked in light blue. 11-mer repeats are underlined. Amino acid numbers are displayed at right. (**B**) Human synuclein structures obtained by Pymol. αS full-length protein structure was obtained from PDB (Protein Data Bank) with the accession code 1XQ8 (https://doi.org/10.2210/pdb1XQ8/pdb (accessed on 19 April 2023)), the unique membrane-bound structure of synuclein family known. The UniProt accession codes Q16143 and Q6FHG5 were used to predict βS and γS full-length protein structures using AlphaFold that draw on structural models from previously determined structures (meaning that these models are not the most accurate) and obtained finally in PDB format, which was used then to exemplify βS and γS structure in Pymol. Different regions of the proteins are highlighted in colors, according to (**A**). (**C**) Evolutionary tree of synucleins. αS, βS, and γS found in different branches of jawed vertebrates are shown. As synucleins were not reported in Caudata (amphibians) and Sphenodon (reptilia) genders, they are not represented. (**D**) Mammal synuclein genomic tree. The common node between αS (pink) and βS (green) is shown in blue. The γS (violet) clade is shown in black. Homo Sapiens taxa are highlighted in red. FASTA files were downloaded from NCBI. Sequence alignment was generated with Molecular Evolutionary Genetics Analysis software (https://www.megasoftware.net/ (accessed on 16 April 2023)). The 311 sequences aligned were uploaded to the IQTREE WEB SERVER (http://iqtree.cibiv.univie.ac.at/ (accessed on 21 April 2023)); the best fit model according to AICc (Second-order Akaike’s information criterion) JTT+G4 was used. The tree was generated using FigTree software (http://tree.bio.ed.ac.uk/software/figtree/ (accessed on 22 April 2023)). (**E**,**F**) Protein (**E**) and mRNA (**F**) synucleins expression in human tissues. Expression information was obtained from The Human Protein Atlas (https://www.proteinatlas.org/ (accessed on 10 April 2023)) and eighteen tissues were selected for plotting. Neuronal tissues are highlighted in violet. H: high; M: medium; L: low; ND: not detected; nTPM: normalized transcript per million.

The amino acid sequence of synucleins (127–140 amino acids) is generally divided into three main regions: N-terminus, nonamyloid component (NAC) region, and C-terminus. Synucleins share significant sequence homology at the N-terminal region, while their C-termini are specific for each member of the family (Figure 1A).

The N-terminal part of synucleins is highly conserved among the three members and is responsible for their lipid-binding properties. The αS N-terminal region is presumed to form one or two α-helices when interacting with the lipid bilayer of membranes [14]. Similar events were described for β and γS, as both proteins are also referred to as IDPs, but upon binding to surfactants or lipids, they rearrange into predominantly a two-α-helix conformation (Figure 1B). It was reported that the αS N-terminal region binds synaptic vesicle membranes, showing a binding preference for highly curved membranes. Although βS and γS share this α-helical lipid-binding motif with αS, they reveal a reduced binding affinity towards membranes [15]. Notably, the most important feature of αS membrane interactions is its incredible plasticity that allows binding to different membrane types (in terms of curvature but also lipid composition, charge, phase, etc.), which translates into different structures. The fact that αS is able to adapt to different membranes (especially vesicles versus plasma membrane) likely contributes to its function [16,17,18,19].

Synucleins’ central core is commonly called the NAC region, because amino acid positions 61–95 in αS were identified as the “non-amyloid β component” found in amyloid plaques associated with Alzheimer’s disease. Several reports point to this region as the highly amyloidogenic part of the molecule, promoting the formation of β-amyloid plaques in vivo [20,21]. The absence of most of the NAC region in βS (Figure 1A,B) is the main determinant for its inability to form amyloid fibrils under physiological conditions. On the other hand, it was described that γS is prone to aggregate into small, soluble oligomers in solution and, upon oxidation of methionine 38, into larger aggregates [22,23].

The C-terminal domain of synucleins does not form part of the amyloid core region or affect the membrane binding ability of this family of proteins. The most remarkable characteristic of this region is its negative charge content, but the role of this protein domain is less understood and controversial. It was proposed that it could be involved in metal binding (calcium, copper, iron, and possibly other metals), but the most putative function for this region is to mediate protein–protein interactions. Supporting this hypothesis, the C-terminal region contains sites for post-translational modifications in all members of this family of proteins, including serine and tyrosine phosphorylation, which can modulate protein interactions [24]. In comparison, γS has a relatively shorter C-terminal domain with fewer acidic residues (Figure 1A,B).

A conserved feature for the three synucleins is the imperfect 11-mer repeat, with the predominant KTKEGV consensus sequence (Figure 1A). The 11-amino acid repeat spans seven times in αS and γS and six times in βS, throughout the N-terminus and NAC region, with slight differences. These repeats were associated with reversible lipid binding, oligomer stabilization, and aggregation [25].

Primary structural analysis of synucleins reveals that αS and βS are more closely related to each other than to γS, although three members of this family have been found in all vertebrates (Figure 1C,D) [26]. To date, no synuclein counterpart was identified in invertebrates, indicating that these proteins are vertebrate-specific. A more refined analysis indicates that the number of members of this family may be different among vertebrates. While all members are present in mammals and birds, this varies in fish depending on the species.

## 3. Synuclein Expression and Physiological Roles

Synucleins are abundant proteins that are mainly found in neural tissue (up to 0.1% of total brain proteins by some estimates) and, to a lesser degree, in red blood cells. In the brain, the αS protein is mainly detected in the cerebral cortex and cerebellum (Figure 1E), although according to “The Human Protein Atlas”, SNCA transcripts are found also in the hippocampus, amygdala, and thalamus among other parts (Figure 1F) [27,28]. Outside the brain, high expression of αS can be observed in bone marrow, kidney, skin, colon, and other tissues. βS brain expression partially correlates with αS, although high levels of this protein are detected in the hippocampus and caudate. Outside the brain, low levels of βS can be found in the kidney and intestines, although SNCB transcripts can also be detected in retinal tissue (Figure 1E,F).

As is the case at the structural level, γS is the most divergent member regarding expression pattern. The γS protein can be detected in the brain at the cerebral cortex, cerebellum, and hippocampus, while expression of this member can also be found in the adrenal gland, bladder, lung, breast, skin, colon, and other organs (Figure 1E,F).

Although synucleins have been well-studied in the context of neurodegeneration and cancer, a clear biological function for synuclein proteins remains poorly understood. As mentioned, the three members of this family bind curved lipid membranes and are involved in the regulation of synaptic vesicle endocytosis [27,28,29]. αS maintains neurotransmitter release by regulating synaptic vesicle pools at the synapse [30] assisting SNARE-complex assembly. It was also suggested that αS maintains normal synaptic function during aging [30]. Nevertheless, almost no clear function was described for βS and γS in the brain, despite their involvement in neurodegenerative diseases [31,32,33]. It was suggested that βS modulates cell survival, metal levels, and dopamine uptake and decreases αS aggregation [34,35,36]. γS physiological function is still even more elusive, but it was proposed that this protein influences neurofilament network integrity and chaperones retinal photoreceptor cells [37,38].

Besides synapses, αS participates in the physiology of other cellular organelles, such as mitochondria, by interacting with mitochondrial proteins such as respiratory chain complexes and ATP synthase and promoting the expression of Miro proteins, which connect mitochondria to microtubules [39]. It was also described that αS interacts with cytoskeletal components and nuclear components [40,41]. αS also has physiological roles in nonneuronal cells such as blood cells, having structural functions and metabolic activities [42].

## 4. Synucleins Are Cancer-Related Proteins

**Figure 2:**
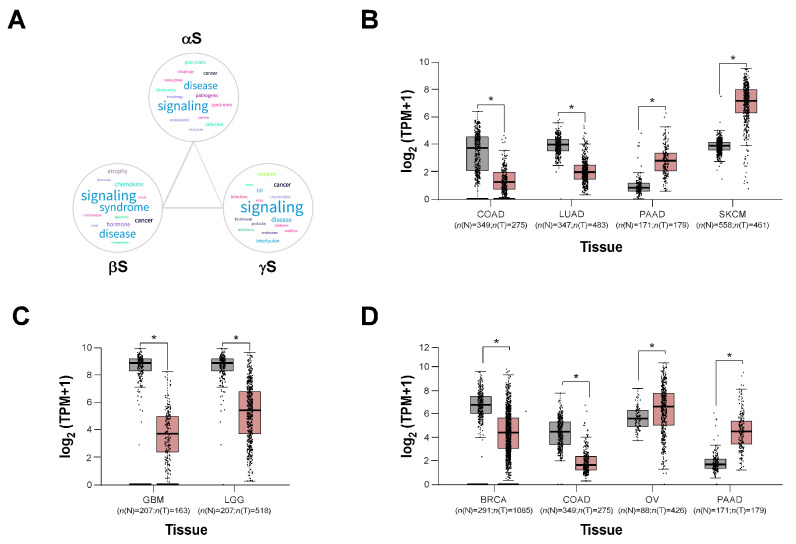
Synuclein expression in cancer. (**A**) To identify synuclein-associated gene clusters, a search was conducted in the NCBI database, limited to Homo sapiens genes. A total of 323, 22, and 49 genes related to αS, βS, and γS, respectively, available in the NCBI database (accessed on 27 April 2023), were downloaded. Using these gene clusters, an enrichment analysis was performed, and a word cloud was generated to display the most common terms found in the results. Word size in the clouds is proportional to the frequency of occurrence in the over-representation analysis (*p* > 0.05), and the thickness of the lines connecting clouds represents the number of shared terms. (**B**–**D**) Expression level of synucleins in tumor samples and noncancerous (normal) samples through GEPIA2 database (http://gepia2.cancer-pku.cn/ (accessed on 10 April 2023)). Light gray represents normal tissues (N) and light pink tumor tissues (T). The expression levels on the Y axis are expressed as log2(TPM + 1), TPM: transcript per million. The statistical analysis performed is *t*-test. * *p* < 0.01. (COAD: Colon adenocarcinoma; LUAD: Lung adenocarcinoma; PAAD: Pancreatic adenocarcinoma; SKCM: Skin Cutaneous Melanoma; GBM: Glioblastoma multiforme; LGG: Brain Lower Grade Glioma; BRCA: Breast invasive carcinoma; and OV: Ovarian serous cystadenocarcinoma). (*n* = number).

Enrichment analysis of genes associated with αS, βS, and γS clearly indicates that all members of this family are involved in cell signaling processes associated with disease development and particularly related to cancer (Figure 2A). This connection to cancer was clearly established for γS [6,43,44,45], but more recently, a putative role in this pathology was described for the other two members.

The first report of the involvement of synucleins in cancer was in 1997, at the time the third member of this family was discovered [6] (Table 1). Two years after discovery, γS was proposed to stimulate breast cancer invasion and metastasis [45]. In 2000, Bruening et al. reported the expression of βS and γS in stage III/IV breast ductal carcinomas and αS, βS, and γS in ovarian carcinomas [46]. At that time, they suggested γS as a putative target for cancer therapy. The same year, two different studies identified the expression of αS in brain tumors showing neuronal or mixed neuronal/glial differentiation [47,48]. In 2001, γS was described as a centrosome-associated protein in retinoblastoma, involved in signal transduction and cell cycle progression [49], and the next year it was described for this member to control cancer cell survival and chemotherapy resistance [50]. In contrast with the pro-tumorigenic role proposed for γS, Zhou et al. suggested in 2003 a negative regulative role for this synuclein in the development of esophageal squamous cell carcinoma [51]. That same year, Fung et al. determined expression of γS in high-grade glial tumors and αS/βS in a high percent of medulloblastomas, but no association between synuclein expression and tumor aggressiveness was established [52]. Following these first reports, many studies pointed to exploring the role of γS in different tumor types such as pancreatic adenocarcinoma [53], gastric cancer [54], bladder cancer [55], and cervical, colon, lung, and prostate cancer [43]. During 2008 and 2009, Ye et al. reported the connection between γS expression and colorectal cancer progression and also explored the expression of the other members of the family in this tumor type, suggesting that co-expression of γS with αS or βS could increase sensitivity to predict advanced stage or lymph node invasion in this tumor type [56,57]. Almost the same γS expression was associated with uterine papillary serous carcinoma [58]. In 2010, Matsuo and Kamitani described the expression of αS in melanoma, suggesting that αS may be the key to understanding epidemiological studies reporting the co-occurrence of melanoma and Parkinson’s disease [59]. αS and βS were proposed as expression markers for specific leukemias by Maitta et al. in 2011 [60]. More recently (2012–2015), new roles for γS were described in endometrial adenocarcinoma [61], gallbladder cancer [62], and oral squamous cell carcinoma [63].

It is clear that many reports link synuclein expression with different types of tumors. Exploring databases recruiting RNA sequencing expression data of tumors and normal samples [68], it is possible to extend these observations. An increased expression of αS in melanoma compared to normal tissue (Figure 2B) correlates perfectly with previous reports [59,69]. This is also the case for pancreatic adenocarcinoma, where an incremental expression was recently described [70]. However, there is a significant decrease in expression for this member of the family in colon adenocarcinoma in contrast with the increased protein level reported in colorectal cancer [57]. Interestingly, SNCA expression in lung adenocarcinoma is lower than in normal tissue, and high αS expression is related to immune infiltration and a better prognosis [71], supporting the negative association reported between Parkinson’s disease and lung cancer [72].

There are no RNA sequencing expression data for the status of βS in medulloblastoma, although protein expression was reported [52]. Nevertheless, transcriptional levels of βS significantly decrease in gliomas and glioblastomas compared to normal tissue (Figure 2B).

The connection between γS and cancer was the first described for a member of this family. As mentioned, γS was initially named as BCSG1 as a result of differential cDNA sequencing studies to identify genes differentially expressed in normal breast compared to breast cancer [6]. Similar results were observed at the protein level by immunostaining of normal and breast cancer tissues [73]. However, RNA sequencing expression data of breast tumors and normal samples suggest a reduction in transcripts for SNCG in this type of cancer (Figure 2B). A similar result can be observed for colon adenocarcinoma, in spite of the reports describing increased γS protein levels in colorectal cancer compared to normal tissue [74,75]. These disparities may reflect the stabilization of this protein in these types of tumors (maybe by post-translational modifications or accumulation of stable high molecular species) or a more efficient translation of RNA. It is also important to note that transcriptional levels in these studies are not related to any specific cell type within the tumor which can account for discrepancies. As reported for protein levels, γS transcripts were significantly increased in ovarian and pancreatic carcinomas (Figure 2B).

## 5. Synuclein Regulation and Post-Translational Modifications

Expression of synucleins is regulated at different levels. αS expression is regulated by various transcription factors such as GATA-1/2, TRIM32, p21, and p27 by direct binding to the promotor region of SNCA [76]. αS expression is modulated by growth factors (nerve growth factor and basic fibroblast growth factor) via MAPK/ERK and PI3K pathways [77], the β2-adrenoreceptor [78], and by dopamine [79].

βS transcriptional regulation was not studied in detail, but tissue distribution data indicate a close similarity of expression and regulation patterns with αS [80]. It was reported that βS expression could be controlled at the transcriptional level by binding of MTF-1 (Metal Transcription Factor-1) to metal response elements at the promoter [81].

γS is overexpressed in a variety of invasive and metastatic cancers and is regulated by multiple transcriptional mechanisms. Overexpression of SNCG in cancer cells may be due to aberrant demethylation of CpG islands within the promoter, AP1 transactivation, and insulin-like growth factor signaling [54,82,83]. It was also reported that TGF-β induces SNCG expression by Smad-Twist1 axis [84].

Synucleins are not only regulated at the transcriptional level, but they are also substantially post-translationally modified. Synucleins’ post-translational modifications (PTMs) may be critical to modulating proteins’ normal and pathophysiological functions and to directing them to different cellular compartments. Several PTMs were described to modulate αS propensity to aggregate by triggering conformational changes, such as phosphorylation, ubiquitination (mono-, di-, and tri-ubiquitination), acetylation, nitration (all four tyrosine residues), and SUMOylation. Particularly, phosphorylation at serine 129 can be detected in blood and it was suggested as a potentially useful biomarker for Parkinson’s disease [85]. Several kinases were demonstrated to be responsible for phosphorylation at this position, including casein kinases I and II, G protein-coupled receptor kinase 2 (GRK2) LRRK2, and PLK, but phosphorylation at other αS amino acids was also reported (S87, Y125, Y133, and Y136) [86].

βS is modified by β-N-acetylglucosamine linked to hydroxyl groups in serine and threonine [87], but this PTM is specific for βS and not αS. βS is also phosphorylated/dephosphorylated at serine residues by polo-like kinase 1 and 3 and PP2A, respectively [35].

Surprisingly, the most studied PTM for γS is its oxidation at methionine 38, which facilitates the formation of aggregates and deposits and was detected in aberrant inclusions in the amygdala of patients with dementia with Lewy bodies, colocalizing with serine 29-phosphorylated αS [88]. It has been proposed that γS oxidation at methionine 38 and tyrosine 39, two of the most easily oxidized residues, allows γS to seed the aggregation of αS [23].

## 6. Synuclein Aggregation and Cancer

αS oligomerization and aggregation were strongly studied in the context of neurogenerative diseases [32,80,88]. As mentioned, γS is also able to form high-molecular-weight fibrils and aggregates and it was also proposed for γS that it could be secreted by exosomes, be transmitted to other cells, and promote aggregation of intracellular proteins in a prion-like manner, as described for αS [23]. Although those events were described in the context of neurodegeneration, several studies suggest a link between protein aggregation and cancer. For example, it was described that both wild type and mutant p53 proteins show kinetics of aggregation and fibrillar morphology that resemble those of classical amyloidogenic proteins, as αS [89,90], suggesting that p53-mutant cancers may be a class of protein aggregation diseases.

Like αS and γS, p53 was described to be transmitted between cells in a prion-like mechanistic fashion [91]. After the discovery of p53 aggregation, other potentially cancer-related proteins were shown to aggregate, such as PTEN, p63, and p71 [92,93]. The question of the impact of protein aggregation in cancer is a research field currently growing, as new reports are connecting aggregation patterns with tumor treatment resistance, tumor progression, and metastasis development [94,95,96].

It was also recently described that αS expression in melanoma is associated with the presence of high-molecular-weight species of this protein, and that treatment with aggregation-inhibiting compounds prevents tumor growth [69], suggesting a key role for this synuclein in melanoma progression, mainly related to autophagy. Additionally, knocking out the αS gene in SKMel28 melanoma cells suppressed tumor growth and promoted dysregulation of cellular iron metabolism [97]. Finally, Dean and Lee demonstrated last year that αS localizes in melanosomes, where it modulates Pmel17 aggregation affecting melanosome maturation and melanin production [98].

To date, no reports addressed the status of oligomerization/aggregation of γS in tumors. However, it is well-described for this protein that it is secreted from tumor cells, and elevated γS levels were especially reported in advanced stages of the pathology.

Although not in cancer, it was proposed in glaucoma that γS oligomeric/aggregated forms could enter the bloodstream, generating autoantibodies [99]. The dynamic intracellular localization of γS and its ability to be transmitted from one cell to another suggest that more implications for this protein in cancer may appear soon.

## 7. Synuclein-Controlled Pathways in Cancer

**Figure 3:**
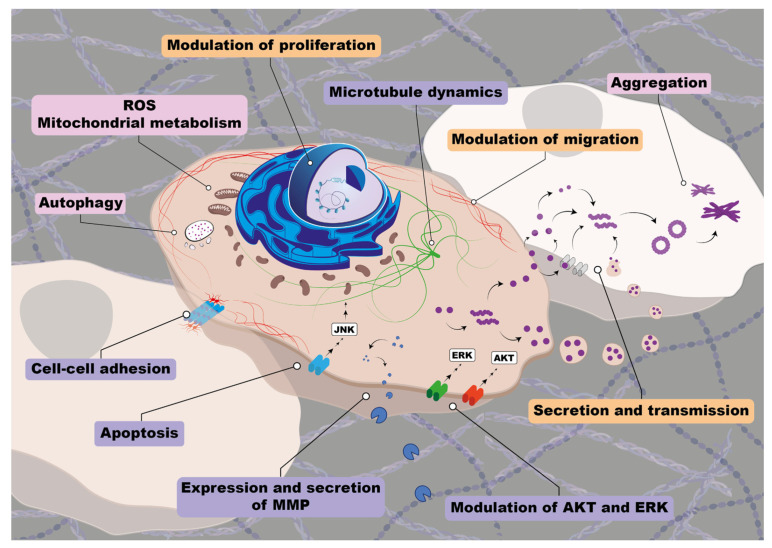
Synucleins are involved in several cancer-related cellular processes. Graphic scheme illustrating key cancer-related cellular processes described for synucleins. The figure shows the main processes involved in a central cell and two neighbors. Light pink boxes represent processes involving αS; light violet boxes represent γS processes; light orange boxes represent processes in which both proteins are involved. αS involvement in autophagy, mitochondrial metabolism, and the generation of ROS are well-established. Additionally, different aggregation states of αS play important roles in cancer. γS levels in tumor cells regulate microtubule dynamics, cell–cell adhesion, apoptosis, and influence signaling pathways involving ERK and AKT. Both synucleins were reported to be involved in cell proliferation and migration. In addition, both proteins can be secreted and transmitted to neighboring cells as monomer or oligomer through different mechanisms (by exosomes release, cross-cell membrane by membrane diffusion, and/or attaching proteins that function as membrane receptors), inducing the aggregation and accumulation of cytosolic synuclein in the proximal cell via prion-like properties, which could have important implications in cancer progression. Depicted organelles and structures include mitochondria, autolysosomes, exosomes, membrane proteins, adhesion proteins, and the cytoskeleton (tubulin and actin filaments). (MMP: extracellular matrix metalloproteinases, ROS: mitochondrial reactive oxygen species).

Many efforts have been directed to understand the role of synucleins in cancer during recent decades. From these studies it seems clear that synucleins play roles at different steps leading to tumor development and progression (Figure 3).

αS levels were reported to affect cell cycle progression and proliferation in osteosarcoma models, affecting tumor differentiation by downregulating proteasome and PKC and upregulating lysosomal activity [64]. It was also described that melanoma and mammary carcinoma cells can uptake exogenously added αS, which promotes in vitro proliferation of those cells [65]. Upregulation of αS was also proposed to contribute to phenotype aggressiveness in meningiomas, affecting proliferation, apoptosis, migration, and invasion of cells by modulating the AKT/mTOR pathway [66]. In melanoma cells, αS was also proposed to modulate cell proliferation by interfering with iron metabolism [97]. The same study also showed in a mouse model that depletion of αS in tumors promotes apoptosis, linking this increase in cell death to high levels of ferric iron. However, Turriani et al. [69] suggested that proliferation of melanoma cells is modulated by αS aggregation, as treatment with oligomer modulators inhibited melanoma cell proliferation and increased apoptosis through dysregulation of cell autophagy. Interestingly, both excess and deficiency of iron can lead to cellular stress, affecting autophagic pathways. It was also demonstrated in neuron cells that iron promotes αS aggregation and transmission by inhibiting autophagosome–lysosome fusion, affecting AKT/mTORC1 signaling [100].

The involvement of AKT/mTOR pathway control by αS was also described for lung adenocarcinoma cells. However, in this context, increased expression of αS inhibited proliferation of pulmonary cells, decreased PI3K levels, and prevented AKT and mTOR phosphorylation [71]. It was also reported that αS inhibits bladder cancer cell proliferation by arresting the cell cycle via upregulation of p53 expression mediated by DNA damage [101]. To support the idea that αS proliferative control strongly depends on the cellular context, it was reported that αS over-expression on PC12 cells enhanced proliferation by increasing cyclin B levels and ERK1/2 phosphorylation and downregulating retinoblastoma [102].

EMT, migration, and invasion are pro-tumoral processes modulated by actin and tubulin cytoskeleton. Several reports described αS interaction with actin and tubulin in the context of neurodegeneration [41,103]; therefore, it is possible that more insights regarding the role of αS in cytoskeleton dynamics will be achieved in the near future.

Other processes related to cancer reported for αS in neurodegenerative models that could play key roles in cancer include its ability to go to the nucleus. Studies based on in vitro and in vivo models suggest αS interacts with DNA and histones and regulates transcription and DNA repair [104]. Still, the role of nuclear αS in tumor cells needs to be further addressed. Furthermore, the impact of αS on mitochondrial energetics and dynamics in tumors needs to be explored in detail, as many reports proposed roles for this protein in mitochondrial oxidative phosphorylation, membrane potential, and homeostasis [105].

So far, there are few reports regarding mechanisms of action for βS in cancer. As protective roles have been attributed to this protein in neurodegeneration (mainly preventing αS aggregation), it would be possible to think that βS could directly or indirectly interfere with pathways such as ERK and PI3K-AKT described for αS both in cancer and neurodegenerative scenarios.

By contrast, several molecular implications were assigned for γS in cancer (Figure 3). It was reported in breast and ovarian cancers that γS over-expression leads to constitutive activation of ERK1/2 and downregulation of JNK1 and that γS promotes resistance to the chemotherapeutic drugs paclitaxel and vinblastine [50]. Furthermore, the interaction of γS with the mitotic checkpoint protein BubR1 in breast cancer is well-documented, leading to mitotic checkpoint compromise through BubR1 inactivation [106]. Furthermore, γS confers cellular resistance to anti-microtubule drugs by interfering with mitotic checkpoint control. In fact, a γS-targeting peptide (ANK) was reported to enhance the sensitivity of breast cancer cells to antimicrotubule drugs [107].

γS was also described to act as an androgen receptor co-activator in prostate cancer, modulating cell cycle progression, proliferation, migration, and invasion [108].

The relation of synucleins with AKT/mTOR pathways is not only restricted to αS, as it was reported that γS binds to the AKT kinase domain, promoting its phosphorylation in non-small cell lung cancer models. In this tumor type, γS promotes cell survival and proliferation by AKT activation, playing a leading role in this pathogenesis [109]. Contrary to what we mentioned for αS, all the reports connecting γS with ERK or AKT pathways indicate that this family member promotes activation of these pathways including gastric [110], cervical [111], ovarian [112], and endometrial cancers [113]. Furthermore, in all the tumor types in which it was explored, γS downmodulates JNK, leading to increased survival and evading apoptosis.

γS expression is directly related to EMT, invasion, and development of metastasis. It is reported that γS favors the expression of metalloproteinases 2 and 9 in retinoblastoma and breast, bladder, cervical, and liver cancers [84,114,115], and it promotes cell motility by regulating Rho GTPases in breast and ovarian cancers [84,116].

Another interesting mechanistic fact associated with γS expression in cancer is related to its secretion. It is documented for colon and breast cancer that γS secretion promotes an aggressive phenotype of cancer cells, favoring invasion [117].

## 8. Synucleins as Cancer Biomarkers

Understanding the characteristics of a tumor allows us to personalize treatments for that particular cancer. The search for cancer biomarkers has grown in recent years as they are used to reflect the incidence and outcome of cancer, but also the effects of treatments or interventions. Thus, cancer biomarkers are normally molecular indicators of cancer susceptibility/risk, occurrence/monitoring of cancer or patient outcome, and they can be detected in biopsy samples or, more interestingly, through non-invasive methods of analyzing blood, saliva, urine, etc. The most relevant property for a biomarker is its usefulness to optimize decisions in clinical practice.

Undoubtedly, αS has been suggested as a biomarker for Parkinson’s disease diagnosis by many studies [85]. However, can synucleins be used as cancer biomarkers? In fact, although more studies should be carried out, they can potentially be good candidates.

It was established that γS has predictive and prognostic values in various types of cancer and it can be used as a stage-specific marker in several tumors [118]. Abnormal γS expression has been related to tumor development, promoting tumor progression and metastasis. The use of γS as a tumor progression biomarker arises from studies detecting serum γS in a high percentage of pancreatic adenocarcinomas, while no presence of this protein was detected in healthy controls [53,55].

**Figure 4:**
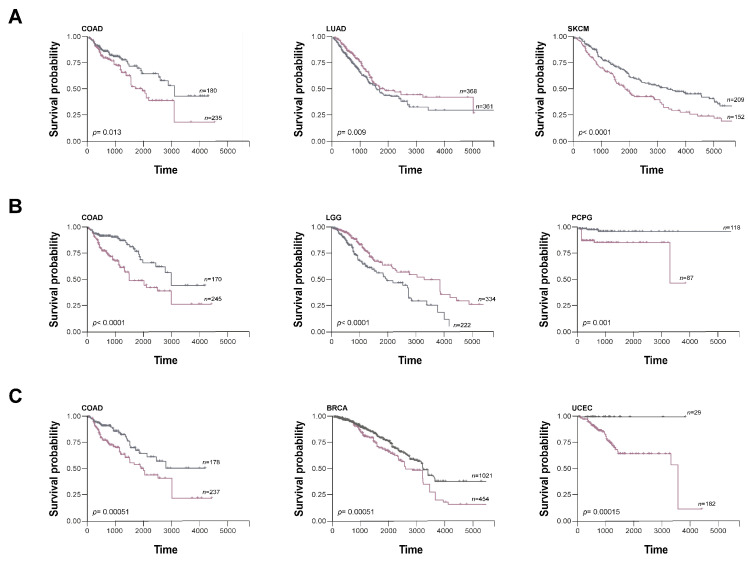
Correlation between synuclein expression and overall survival. (**A**–**C**) Association between synuclein expression and overall survival (OS) using TCGA (The Cancer Genome Atlas) mRNA expression datasets for different tumors. Kaplan–Meier curves for OS of cancer patients with low (gray line) versus high (violet line) expressions of αS (**A**), βS (**B**), and γS (**C**) were generated using Survminer R package (version 0.4.9) (*p* < 0.05) and compared by log-rank tests. The number of patients for each case is described in the figure (*n*). The *x*-axis depicts time in days. (COAD: Colon adenocarcinoma; LUAD: Lung adenocarcinoma; SKCM: Skin Cutaneous Melanoma; LGG: Brain Lower Grade Glioma; BRCA: Breast invasive carcinoma; PCPG: Pheochromocytoma and Paraganglioma; UCEC: Cervical squamous cell carcinoma and endocervical adenocarcinoma).

As mentioned before, Ye et al. corroborated synuclein expression in colorectal cancer by IHC, predicting that γS is a good marker for cancer progression, but simultaneous detection of αS/γS or βS/γS predicted advanced stage and lymph node invasion [57]. Accordingly, analysis of transcriptional levels of αS, βS, and γS in colon adenocarcinoma indicates a clear correlation between high transcriptional levels for genes coding these proteins with a poor prognosis (Figure 4). Similarly, it was described that γS in breast cancer is related to poor prognosis [73] and analysis of SNCG transcriptional levels in breast cancer shows that they are significantly associated with poor outcome.

Increased expression of αS in lung adenocarcinoma was proposed as a good prognostic biomarker as it directly correlates with increased immune infiltration and better prognosis [71]. In line with this report, high SNCA expression correlates with increased survival, and the inverse association is observed in melanoma, in agreement with the pro-tumoral role observed for αS in this tumor type. In addition, αS was recently proposed as a biomarker for Group 4 medulloblastoma [67], the larger subtype (up to 30%) with the least characterized molecular pathogenesis of this tumor type.

βS expression could also be used as a prognostic marker in glioma and pheochromocytoma/paraganglioma, as SNCB expression is associated with good and worse prognosis, respectively.

Nevertheless, it is important to have in mind that tumor mRNA levels do not always correlate with tumor protein levels or even with the abundancy of protein in fluids. As an example, expression of γS in bladder carcinoma was proposed to be a good marker to predict recurrence, but not a reliable marker for staging or prediction of survival rate [119]. Indeed, high transcriptional SNCG levels are associated with good prognosis in bladder cancer (data not shown).

During recent decades, several studies associated γS levels with poor outcome in endometrial adenocarcinoma [61,113], which correlates with a worse prognosis for uterine corpus endometrial carcinoma patients with high levels of SNCG transcripts (Figure 4). It has also been proposed that γS could be a prognostic marker for tumor cell migration in biliary carcinomas [120].

As synucleins are detectable in fluids such as blood, saliva, urine and others, further comprehension of the potential role of these proteins as biomarkers stands as a very promising field to improve diagnosis, progression, and monitoring of patients through reliable non-invasive methods.

## 9. Conclusions

Cancer is one of the leading causes of death worldwide. On the other hand, population longevity associated with an increase in life expectancy brings an increasing risk for neurodegenerative disorders. Both pathologies affect millions of people, severely compromising the quality and expectation of life and representing one of the most chronic diseases.

Cancer and neurodegeneration are associated with opposite ends, as one is related to cell proliferation and cell death resistance, while the other is directly linked to premature cell death. Nevertheless, these two diseases are not so distant.

In this work, we focused on synucleins, a family of small proteins that could represent a link between cancer and neurodegeneration. Reports describing the involvement of synucleins in cancer increased rapidly during recent decades, suggesting that these proteins, initially associated with neurodegeneration, play crucial roles in cancer progression and many studies have provided conclusive evidence to support the idea that this family of proteins is involved in cell signaling processes related to cancer development. The importance of synucleins in cancer is such that they have been proposed as relevant biomarkers for several tumor types, showing a potential to enhance the accuracy of diagnosis, tracking the progression of disease, and monitoring patients using non-invasive techniques.

Interestingly, as the physiological function of these proteins is not fully understood, knowledge gained about synucleins in one field could lead to advances in the other, and thus feed off each other.

## Figures and Tables

**Table 1:** Selected reports describing the involvement of synucleins in cancer.

Synucleins	Year	Tumor Type	Type of Study	Reference
γS	1997	Breast	Patients	[6]
γS	1999	Breast	Cell lines	[45]
βS, γS	2000	Breast, Ovarian	Cell lines and patients	[46]
αS	2000	Ganglioglioma, Ganglioneuroblastoma	Patients	[47]
αS	2000	Medulloblastoma, Pineocytoma, Pineoblastoma	Patients	[48]
γS	2001	Retinoblastoma	Cell lines	[49]
γS	2001	Ovarian	Cell lines	[50]
γS	2003	Esophageal	Cell lines and patients	[51]
αS, βS	2003	Glial, Medulloblastoma	Patients	[52]
γS	2004	Pancreatic	Cell lines and patients	[53]
γS	2004	Gastric	Cell lines and patients	[54]
γS	2004	Bladder	Patients	[55]
γS	2005	Prostate, Cervical, Colon, Lung	Patients	[43]
αS	2007	Osteosarcoma	Cell lines	[64]
γS	2008	Colon	Cell lines and patients	[56]
γS	2009	Uterine serous papillary carcinoma	Cell lines and patients	[58]
αS, βS, γS	2010	Colon	Cell lines and patients	[57]
αS	2010	Melanoma	Cell lines and patients	[59]
αS, βS	2011	Leukemia	Cell lines and patients	[60]
αS	2011	Melanoma, Breast, Lung	Cell lines and mice	[65]
γS	2012	Endometrium	Patients	[61]
γS	2016	Squamous cell carcinoma of the oral cavity	Cell lines and patients	[63]
αS	2016	Meningioma	Cell lines and patients	[66]
γS	2021	Biliary tract	Cell lines and patients	[67]

## Data Availability

Not applicable.
